# Proton pencil beam scanning radiotherapy in the postoperative treatment of p16 positive squamous cell tonsillar cancer – evaluation of toxicity and effectivity

**DOI:** 10.1007/s00405-024-08747-1

**Published:** 2024-08-28

**Authors:** Jiří Kubeš, Sarah Al-Hamami, Silvia Sláviková, Pavel Vítek, Alexandra Haas, Kateřina Dědečková, Barbora Ondrová, Michal Andrlik, Matěj Navrátil, Eliška Rotnáglová, Vladimír Vondráček

**Affiliations:** 1 Proton Therapy Center Czech, Budínova 1a, Prague 8, 18000 Czech Republic; 2 Department of Health Care Disciplines and Population Protection, Faculty of Biomedical Engineering, Czech Technical University Prague, Sítná square 3105, Kladno, 272 01 Czech Republic

**Keywords:** Tonsillar cancer, Proton therapy, Radiotherapy, Toxicity, Survival

## Abstract

**Purpose:**

Patients with p16 positive tonsillar cancer (p16 + TC) have an excellent prognosis and long-life expectancy. Deintensification of therapy is a prevalent topic of discussion. Proton radiotherapy is one way to reduce radiation exposure and thus reduce acute and late toxicity. The aim is to evaluate treatment outcomes and toxicity of postoperative treatment with intensity-modulated proton therapy (IMPT).

**Methods:**

Between September 2013 and November 2021, 47 patients with p16 + TC were treated postoperatively with IMPT. Median age was 54.9 (38.2–74.9) years, 31 were males and 16 were females. All patients had squamous cell carcinoma and underwent surgery as a primary treatment. Median dose of radiotherapy was 66 GyE in 33 fractions. Bilateral neck irradiation was used in 39 patients and unilateral in 8. Concomitant chemotherapy was applied in 24 patients.

**Results:**

Median follow-up time was 4.2 (0.15–9.64) years. Five-year overall survival, relapse free survival and local control were 95.7%, 97.8% and 100%. The most common acute toxicities were dermatitis and mucositis, with grade 2 + in 61.7% and 70.2% of patients. No acute percutaneous gastrostomy insertion was necessary and intravenous rehydration was used in 12.8% of patients. The most common late toxicity was grade 1 xerostomia in 70.2% of patients and grade 2 in 10.6% of patients. Subcutaneous fibrosis of grades 2 and 3 occurred in 17.0% and 2.1% of patients, respectively. One patient developed late severe dysphagia and became PEG-dependent.

**Conclusion:**

IMPT for the postoperative treatment of p16 + TC is feasible with excellent efficiency and acceptable acute and late toxicity.

## Introduction

The incidence of tonsillar cancer has doubled in the last 20 years, marking one of the most pronounced surges in cancer prevalence. This escalation is primarily attributed to a significant increase in the proportion of human papillomavirus 16 (HPV16) positive tumours, which currently predominate [1]. HPV16 positive tumours are characterized by patients of a younger demographic with fewer comorbidities, less alcohol and tobacco consumption, and with significantly better disease prognosis [2]. Classically, tonsillar cancer is treated by a combination of surgery, chemotherapy, and radiotherapy. However, concerns regarding high rates of treatment-induced toxicity, such as dysphagia, xerostomia, and long-term impairment of quality of life have prompted the search for a more refined treatment approach [3–6]. Consequentially, the de-escalation of treatment intensity of tonsillar cancer is a subject of major discussion. De-escalation approaches include reducing the total radiotherapy dose, omitting concomitant chemotherapy, or irradiating ipsilateral nodal areas only. Proton therapy presents an additional option for reducing radiation exposure in the head and neck area, by providing a dosimetric advantage over intensity-modulated radiation therapy (IMRT) [7]. The aim of this retrospective study was to evaluate the feasibility, acute and late toxicity and oncological outcome of proton radiotherapy using the IMPT technique in patients indicated for post-operative radiotherapy for p16 + tonsillar cancer.

## Materials and methods

### Patient demographics and characteristics

We searched our institutional database for patients with histologically confirmed p16 + squamous cell tonsillar cancer who underwent radical surgery and were indicated for postoperative radiotherapy, treated between September 2013 to November 2021. Patients who did not have surgery as part of their treatment plan, and those who had recurrent head and neck cancer previously treated with curative radiotherapy were excluded. The study was approved by an institutional ethical committee, and was conducted according to local ethical standards. The search identified 47 patients, and all that were treated with curative intent passed the inclusion criteria. Patients were treated for p16 + squamous cell tonsillar cancer using pencil beam scanning (PBS) proton radiotherapy to the primary tumour bed and bilateral or ipsilateral neck lymph node areas. Demographic and treatment characteristics are shown in Table 1. Patients treated before 2017 were reclassified according to the 8th AJCC edition. All patients provided their written informed consent for therapy and data processing, and treatment was performed in accordance with local standards.

**Table 1:** Demographic and treatment parameters

	*n*	%
Age (mean, y)	54.9 (38.2–74.9)	
Age < 50y	15	31.9
Age > 50y	32	68.1
**Sex**		
male	31	65.0
female (%)	16	35.0
**Histology**		
Spinal cell cancer p16+	47	100,0
**pT stage**		
pT1	20	42.6
pT2	23	48.9
pT3	4	8.5
pT4	0	0
**pN stage**		
pN0	4	8.5
pN1	42	89.4
pN2	1	2.1
pN3	0	0
**Group AJCC stage (8 ed)**		
I	39	83.0
II	8	17.0
III	0	0
IV	0	0
**Surgical margin status**		
R0	24	51.1
R1	22	46.8
R2	1	2.1
**Neck dissection**		0,0
unilateral	45	95.7
bilateral	2	2,3
**Extracapsular extension**		0,0
yes	8	12.8
no	39	87.2
**Radiation dose (GyE)**	66 (62–74)	
**Concurrent chemotherapy**		
Yes	24	51.1
No	23	48.9
**Neck radiotherapy**		
Unilateral	8	17.0
Bilateral	39	83.0

### Immobilization, set-up and planning procedures

Patients were treated using standard five-point immobilization devices (thermoplastic masks, Orfit) in the supine position. Prior to the planning CT, dental treatment was performed to remove metal bridges and replace any amalgam dental fillings with composite fillings to reduce artifacts on CT scans, if it was considered necessary for the purpose of calculating the dose. Computer tomography was used for treatment planning (slices of 2.5 mm) and image registrations with preoperative or planning MRI and/or PET FDG scans were performed before contouring.

### Target volume delineation

The contouring of target volumes and organs at risk was performed using Focal software (Elekta AB, Sweden) or RayStation software (RaySearch, Sweden). Contouring of target volumes was carried out using the same recommendations as for photon radiotherapy. The clinical target volume was defined as the as the pre-surgery tumour volume with a 1 cm margin around it, excluding bones and air cavities. The CTV for lymph nodes encompassed bilateral neck lymphatic areas based on standard recommendations [8, 9]. A 5 mm expansion from the CTV was used to generate the planning target volume (PTV). The following organs at risk (OAR) were contoured: brain, brainstem, temporal lobes, eyes, retinas, lenses, optical nerves, optic chiasma, cochleas, parotid glands, pharyngeal constrictors, oesophagus, larynx, thyroid gland, spinal cord and temporomandibular joints. The skin was not recognized as an OAR until June 2015, after which it was contoured as a critical organ, defined by the intersection of a 2 cm margin around the PTV and a “body wall” structure 4 mm inward from the body’s surface.

### Dose prescription and chemotherapy

**Fig. 1:**
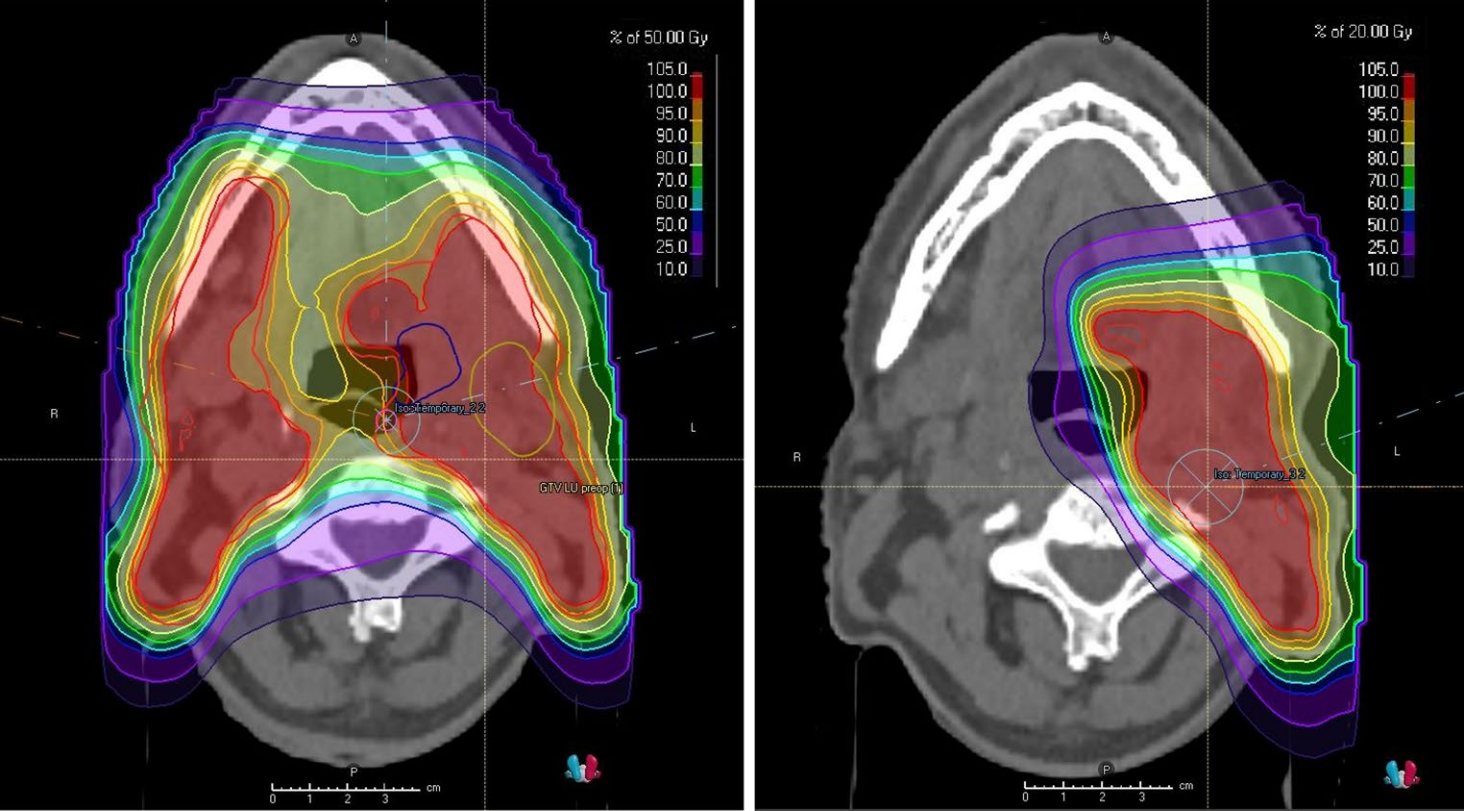
An example of typical dose distribution for postoperative irradiation of the tonsillar bed and bilateral neck region (colour wash with 5% as lower limit). The first phase with bilateral neck lymph nodes irradiation (left) and boost to left tonsillar area (right)

Treatment was performed in two sequential phases – 50 GyE in 25 fractions for the tumour bed and bilateral neck lymph node areas, followed by 16–20 GyE in 8–10 fractions for the primary tumour CTV and involved neck lymph node areas. A higher dose in the 2nd phase of treatment was used in the case of R1-2 resection or for persistent tumours detected on postoperative examinations. An example of dose distribution is shown in Fig. 1. Weekly cisplatin (40 mg/m^2^) was administered based on the decision of the attending physician, according to the associated risk factors (T-stage, grade, number of positive lymph nodes, ECE, R1 resection), or in the case of persistent disease.

### Planning, optimization, robustness

In the treatment planning process, two different planning systems were used. In 2020, a switch was made from the treatment planning system XiO (Elekta, Sweden) to RayStation (RaySearch, Sweden). With XiO, the dose was calculated in Grays (Gy) and conversion to a radiobiologically equivalent dose (GyE) was performed using a factor of 1.1. With RayStation software, the implicit conversion from the physics dose to relative biological effectiveness, i.e. by a factor of 1.1 (RBE) dose was implemented. For patients with bilateral lymph node neck irradiation, the beams were set-up as two oblique lateral fields. In cases where the lower neck lymph nodes or the upper mediastinal lymph nodes were involved, one additional anterior field was used. In cases with unilateral lymph node irradiation, the target volume was treated with one or two oblique beams. At the distal edge, the dose was selectively reduced to account for the increased radiobiological effectiveness at the distal beam fall-off. All treatment plans were carried out using the IMPT technique with a full optimization approach. The number of layers was appropriate to the size of the PTV to avoid ripples in the depth profile. Spot spacing was usually chosen to be 4 mm. No class solution was introduced into the planning process. The dose distribution was measured by a 2D detector at several depths and evaluated using gamma analysis (Δ_Dose_ 3%. Distance to agreement 3 mm) with the acceptance criteria set at 95% of the points with γ < 1. In some cases, an in-house log-based quality assurance (QA) software tool was used for pre-treatment verification.

Treatment plans were also inspected in the mean of robustness evaluation. For the plan, the isocenter was artificially moved by 2 mm in each spatial direction to mimic setup errors and their influence on dose distribution. This approach has been shown to minimally affect the dose to critical organs, if the movement is limited to 2 mm. Such precision is usually well achieved during the set-up of each fraction. Additionally, in order to mimic range uncertainty and to take into account possible imperfectness of the calibration of planning CT, another two sets of plans were generated with 2 mm shifts; first with the CT calibration curve shifted by + 3.5% and second with − 3.5%. Since treatment plans are created in the same way for all patients (in the sense of beam setup, splitting of target volumes and constraint) the robustness evaluation was not performed for all patients. For a typical treatment plan, we created 12 plans to evaluate plan robustness. If more than one of those plans failed to fulfil constraints, the plan was re-examined and changes to the optimization process were made; the cause of an exceeding dose to the critical structure was determined and specific measures were inserted to modify dose distribution in an appropriate way. This approach was used to fine-tune treatment using the “class solution” approach.

### Adaptive replanning

All patients were treated using daily kilovoltage (kV) image guidance. Control computed tomography (CT) scans were performed once weekly with image registration with CT planning and preparation of quality assurance plans in the case of significant change of contours of body or OAR. New plans were created when dose distribution changed, mainly due to changes in patient contours due to weight loss. Limits for dose changes were individual and dependent on the location of the change; however, a change of over 5% of the dose inside the critical organ or target volume triggered the re-planning process. Fifteen patients required replanning one time and three patients had two replans. The remaining patients did not require any replanning.

## Results

### Overall survival, relapse-free survival and locoregional control

**Fig. 2:**
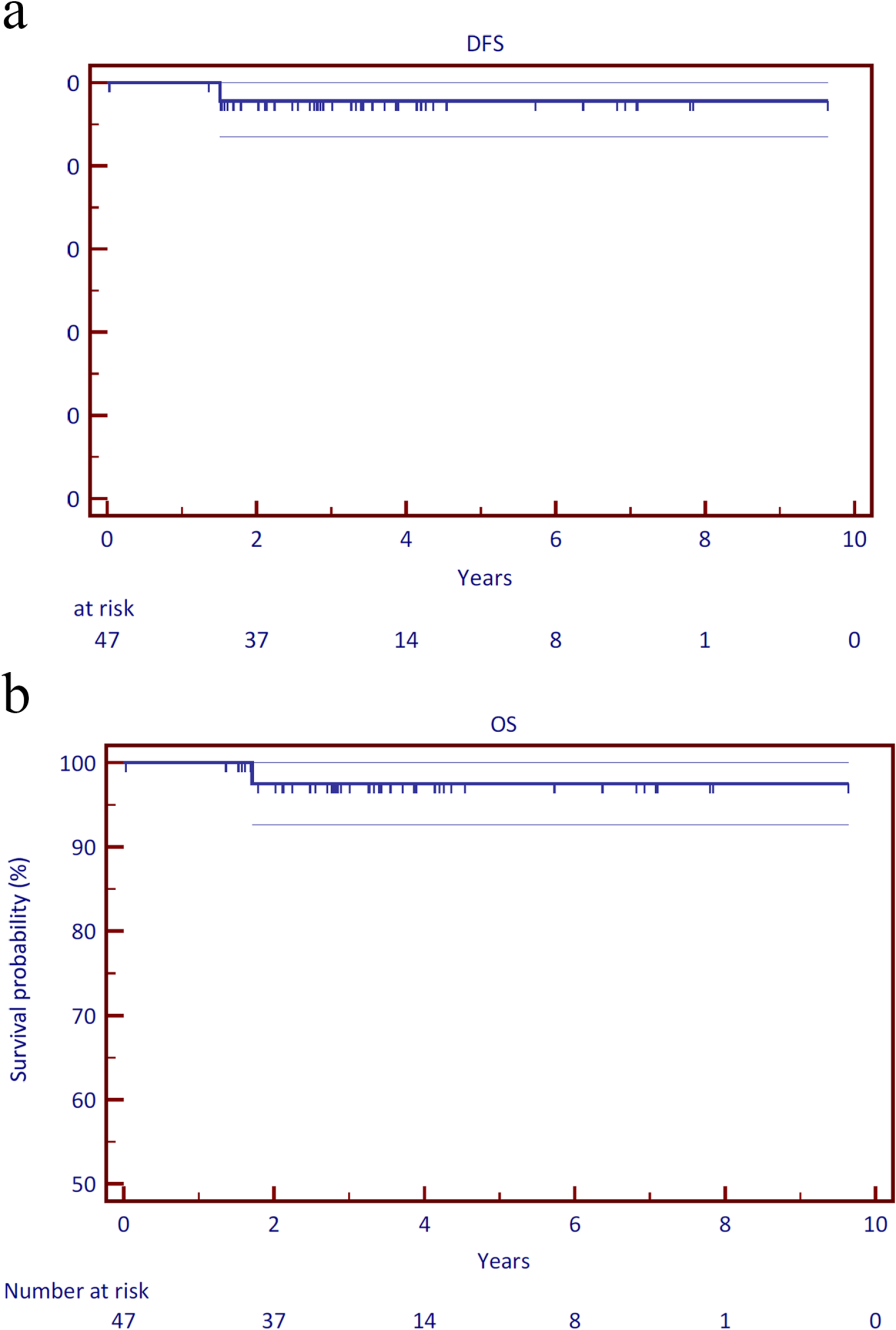
Kaplan-Meier curves for (**a**) overall survival (OS) and (**b**) relapse-free survival (RFS)

The median time for follow-up was 50.4 months (range: 1.8–124.8 months). All patients were treated without interruptions. The three-year and five-year overall survival (OS) rates were both 95.7% (± 3.0%), respectively. Three death events occurred, two before three years and one in the tenth year of follow-up. During the follow-up period, relapse-free survival (RFS) and locoregional control rates were 97.8% (± 2.2%) and 100%, respectively (Fig. 2). Progressive disease was seen in one (2.1%) patient who had atypical distant progression with brain, lung, and soft tissue metastases. Two months after the distant metastases were diagnosed, the patient died of disease progression. One patient deceased due to reasons unrelated to cancer (suicide), and one patient deceased due to unknown reasons. Kaplan-Meier curves for OS and RFS can be found in Fig. 2.

### Acute Toxicity

Acute and late toxicities were evaluated using the Common Terminology Criteria for Adverse Effects CTCAE v5 scale and are shown in Table 2. The most common acute toxicities were radiation dermatitis, mucositis and dysphagia. Dermatitis was a serious problem in patients treated before June 2015, when there was a lack of optimization of treatment plans for skin as a critical organ. Grade 3 dermatitis was no longer observed after the introduction of skin as an OAR. Mucositis leading to eating disorders was observed in up to 75% of patients. However, no patients required acute PEG insertion. Temporary intravenous rehydration was required in only 6 patients (12.8%), while 8 (17.0%) patients used weak opiates and only one (2.1%) patient received strong opiates.

### Late toxicity

Late toxicity was generally mild. The most common problem was mild xerostomia, with grade 1 occurring in 33 (70.2%) patients. This did not significantly affect food intake. Significant subcutaneous fibrosis (grade 2+) developed in 9 (19.1%) patients, all of which had significant (grade 2+) acute skin reactions (skin was not yet an OAR). One patient had a temporary tracheostomy for laryngeal edema. After the edema disappeared, the tracheostomy was removed and at the 5-year post-treatment control, the patient was without significant problems. Two patients (4.3%) were recorded to have temporary trophic defects in the tumour bed after tonsillectomy, osteoradionecrosis was demonstrated in one patient (2.1%), clinically asymptomatic carotid stenosis in one patient (2.1%), upper extremity neuropathy in one patient (2.1%), and hypothyroidism in four patients (8.5%). One patient developed severe dysphagia requiring a PEG due to a trophic soft-tissue defect in the tonsillectomy bed; this patient later on died due to brain metastases and subcutaneous cancer dissemination.

**Table 2:** Acute and late toxicity

CTCAE v. 5	0	1	2	3	NA
**Acute toxicity**					
Mucositis, n (%)	0 (0%)	11 (23.4%)	33 (70.2%)	3 (6.3%)	
Dermatitis, n (%)	0 (0%)	12 (25.5%)	29 (61.7%)	6 (12.7%)	
Dysgeusia, n (%)	14 (29.8%)	27 (57.4%)	5 (10.6%)		1 (2.1%)
Weight loss, n (%)	18 (38.2%)	20 (42.6%)	8 (17.0%)	1 (2.1%)	
	**No**	**Yes**			
Acute PEG insertion, n (%)	47 (100%)	0 (0%)			
Rehydration, n (%)	41 (87.2	6 (12.8%)			
Opioid use, n (%)	39 (83%)	8 (17.0%)			
**Late toxicity**					
Dysphagia	41 (87.2%)	3 (6.4%)	1 (2.1%)	1 (2.1%)	1 (2.1%)
Hoarsness	44 (93.6%)	2 (4.3)	0 (0%)	0 (0%)	1 (2.1%)
Dysgeusia	38 (80.9%)	8 (17.0%)	0 (0%)	0 (0%)	1 (2.1%)
Xerostomia	8 (17.0%)	33 (70.2%)	5 (10.6%)	0 (0%)	1 (2.1%)
Trismus	42 (89.4%)	4 (8.5%)	0 (0%)	0 (0%)	1 (2.1%)
Subcutaneous fibrosis	21 (44.6%)	16 (34.0%)	8 (17.0%)	1 (2.1%)	1 (2.1%)
Dermatitis	38(80.9%)	6 (12.8%)	2 (4.3%)	0 (0%)	1 (2.1%)
	**No**	**Yes**			
PEG dependency	45 (95.7%)	1 (2.1%)			1 (2.1%)

## Discussion

Patients with tonsillar cancer treated with a combination of surgery and adjuvant radiotherapy have a good prognosis in general, especially if the tumour is p16 positive. In a study done by the KROG (Korean Radiation Oncology Group), where p16 status was unknown in the majority of patients, the 5-year DFS for the bilateral nodal radiotherapy group was 94.2% [10]. In a recent study by Ferris RL et al. [11] reporting results of a phase II study of postoperative radiotherapy in p16 positive oropharyngeal cancer, the two-year progression-free survival was 94.9% for the 5 Gy arm and 96% for the 60 Gy arm. With such a high percentage of long-term survival, late toxicity is a crucial factor that must be considered. Therefore, in tonsillar cancer, ways to de-escalate the intensity of treatment are strongly sought after. The most frequently discussed options are dose de-escalation, chemotherapy omission and unilateral adjuvant radiotherapy. Proton radiotherapy is another possibility for de-escalation of treatment intensity.

Proton radiotherapy has dosimetric advantages in the head and neck area. Several dosimetric studies have been published for oropharyngeal carcinoma. Holliday et al. [7] compared IMPT with IMRT and found that IMPT leads to lower mean doses to many OARs, including the anterior and posteriorsections of the oral cavity, middle and inferior pharyngeal constrictor muscles and oesophagus. Apinorasetsethkul et al. [12] compared proton radiotherapy and volumetric modulated arc therapy (VMAT) for oropharyngeal cancer and found that protons offered improved sparing of the oral cavity, contralateral parotid gland, and contralateral submandibular gland. However, sparing of the parotid glands is highly dependent on the arrangement of the fields for PBS.

The integral dose and potential induction of secondary malignancies also plays a role, considering the relatively young age of patients with tonsillar cancer and their long-life expectancy. Jain et al. [13] compared the risk of secondary malignancies after IMPT or IMRT for oropharyngeal cancer using an organ equivalent dose model for the linear-exponential dose-response curve. They found that at a median age of 54 years at the time of treatment, with an average life expectancy of an additional 27 years, 4 excess secondary malignancies per 100 patients could be avoided by treating them with IMPT versus IMRT.

A particular disadvantage of proton radiotherapy is its higher sensitivity to the accuracy of performance and reproducibility of target volumes. In the postoperative setting in patients with tonsillar cancer, i.e., in a group of patients in a good condition enabling surgery, where the tumour has been removed, problems with changes to the target volume, and the need for adaptive radiotherapy with frequent replanning, are eliminated. Higher sensitivity to set-up errors can be addressed by the robust optimization of treatment plans to be more resistant to set-up errors. The feasibility and applicability of this technique in oropharyngeal cancer has been demonstrated by Hague et al. [14]. Our approach to robustness assessment is described in the section concerning treatment planning. In principle, it is not feasible to create fully robust plans without a pro and con evaluation. Using a standardised treatment approach, all treatment plans we used had the same level of robustness. Before the actual treatment technique was introduced into clinical practice, robust evaluation was performed for typical setup.

Treatment results are generally excellent in HPV16 positive oropharyngeal tumors [15, 16]. To this point, our group of patients did not deviate in any way, and the overall survival and relapse-free survival parameters are comparable to other research outcomes. During our follow-up period, metastasis occurred in one patient (97.9%). Similar studies using photon radiotherapy reported an 8% metastasis rate [17] and 7% in a study utilising only unilateral lymph node irradiation [18].

The toxicity of the treatment was mild, but we observed more pronounced cutaneous toxicity with subsequent subcutaneous fibrosis in patients treated at the beginning of the study period. The problem of skin toxicity has been described, for example, in the case of breast cancer [19]. This was due to an approach to planning that did not sufficiently take into account the skin as a critical planning authority. After tightening the parameters for skin as an OAR, this problem was significantly reduced.

Our rates for other acute toxicities such as oral mucositis and weight loss were comparable and, in some cases, better than in photon-based studies. Developing oral mucositis during radiotherapy can be very painful, and can lead to difficulties with eating, leading to malnutrition [20]. Kim et al. [10] reported oral mucositis grades 1, 2 and 3 in 24.3%, 47.1% and 18.6% of their bilaterally irradiated patients, respectively. Chin et al. [17] observed mucositis grades of 1, 2 and 3 in 10.0%, 50.0%, 32.0% of their patients, respectively. While these studies had more patients with no mucositis (10.0% and 8.0%, respectively), we report considerably less patients with grade 3 mucositis. Our study also revealed markedly lower rates of higher-grade weight loss compared to the 38%, 16%, 8%, 38% of photon-treated patients with grades 0, 1, 2 and 3 [17]. The low percentage of tube feeding required was noteworthy. In a photon-based study with 59 patients receiving bilateral IMRT, PEG was used acutely in 39% of patients and long-term in 20% [17]. Additionally, we had markedly lower rates of late dysphagia compared to the 30% of patients that developed grades 1–3 in another study [10].

Proton radiotherapy could achieve maximum potential in unilateral radiotherapy in the adjuvant treatment of HPV16 positive tonsillar cancer. However, this approach is not yet standard, particularly due to fear of a risk of progression to the contralateral nodes. For example, Kim et al. [2] demonstrated the importance of contralateral radiotherapy for N2b stages of the disease (AJCC 7th edition). However, a higher proportion of contralateral relapses did not translate into overall survival. The main advantage of proton radiotherapy in this context is zero dose to the contralateral side of the neck when the ipsilateral area is irradiated. The contralateral side remains therefore intact, and any other procedures, including surgery and subsequent radiotherapy, can be performed as in treatment-naive patients.

## Conclusion

Proton radiotherapy using the pencil beam scanning technique is feasible in postoperative radiotherapy of tonsillar cancer. The oncological results are comparable with photon radiotherapy techniques. Acute and late toxicity is low, with minimal need to introduce PEGs for acute or late dysphagic disorders. We draw attention to the need for dose optimisation for the skin as a critical organ. We see the greatest potential of proton radiotherapy in unilateral neck radiotherapy, which offers a way to de-escalate treatment intensity in HPV16 positive tumours. Limitations of our study include its retrospective nature, which could result in an underestimation of toxicity, as well as the size of the cohort. Our results should be confirmed through more extensive and prospective work. However, we consider the homogeneity of our patient group to be one of the strengths of this study. We believe this summary draws attention to optimal indications for proton therapy with its excellent potential to spare healthy tissues in the case of unilateral disease.

Acknowledgements.

## Data Availability

Anonymized-patient data for this study are available upon request from the corresponding author.
